# Review of "Fundamentals of Clinical Trials" by LM. Friedman, CD. Furberg and DL. DeMets

**DOI:** 10.1186/1475-925X-3-43

**Published:** 2004-11-25

**Authors:** Max E Valentinuzzi

**Affiliations:** 1 Department of Bioengineering, Instituto Superior de Investigaciones Biológicas (INSIBIO), CONICET, Argentina; 2 Universidad Nacional de Tucumán (UNT), Argentina

*Clinical Research *faces perhaps a certain grain of confusion. Often, it is mistakenly reduced to case report. Caveats of even greater risks may be hidden in the secrets of patents and interests of manufacturers covertly promoting their products. Thus, a book clearly stating what a clinical trial is, well located within the scientific frame, appears as a significant contribution enhancing the importance and complexity of XXIst Century human health.

The book is preceded by two editions (1981 and 1985) and well backed by the experience and prestige of its authors. *Clinical Research*, a branch of *Applied Research*, finds in clinical trials the most definitive tool for evaluation of its actual applicability. The book is divided in 19 chapters, each setting up unequivocally the *Fundamental Point *to be treated, as for example the introductory Chapter 1: *A properly planned and executed clinical trial is a powerful experimental technique for assessing the effectiveness of an intervention*. A *clinical trial *compares the effect and value of intervention(s) against a control in human beings. *Intervention *refers either to a drug, a procedure or a technological device, thus implying that biomedical engineering scientists and professionals might be involved in it. *Clinical Research *always presents early stages where the laboratory work is mandatory. This book emphasizes the concept all along, so placing the clinical trial in its proper hierarchical perspective. There is laboratory work during drug, device and/or procedure development, animal studies and early tests in small number of human beings. Case reports, typical of the hospital, find their place as a warning of adverse effects or as a flag of an unexpected beneficial effect.

In Chapter 2 the authors emphasize that a clinical trial must have a primary question. It should be carefully selected, as any subsidiary question must be, too. Otherwise, the investigators may lose the track. Chapter 3 deals with the study population, underlying the importance of unambiguous eligibility criteria, the latter having a direct influence on the recruitment of participants and, thus, on the final number involved in the project (which may end up with not enough data). Chapter 4 outlines the basic study design; it stresses the demand of a control group and the need of randomization for assigning participants to control and intervention groups.

An extremely important subject is the randomization process, essential to avoid any possible bias (Chapter 5). Several forms are presented: simple, blocked and stratified. More elaborate procedures make use of adaptive methods. There are examples and an appendix with an algorithm of general applicability. Bias (a systematic error) is one of the main concerns; thus, a double-blind design is essential (Chapter 6). Chapter 7, *Sample Size*, addresses the question of how many subjects is enough. The trial must have sufficient statistical power to detect differences between groups. Therefore, calculation of sample size with provision for adequate levels of significance is essential. Several situations with their respective examples are presented making of this pages an invaluable tool. Unfortunately, several equations have been printed in such a way that there is confusion on whether some parameters are factors or exponents.

Baseline refers to the status of participants before the start of the intervention (Chapter 8), while Chapter 9 deals with the recruitment of participants. The latter depends on developing a careful plan with multiple strategies. Chapter 10 enters into data collection and quality control, wisely underlining that no study can be better than the quality of its data. Monitoring and even auditing are thus necessary. The history of new medical interventions abounds in sad experiences. Hence, adverse effects, if any at all, must be searched and assessed. Chapter 11 clearly probes into this complex aspect. Sadly, perhaps due to sheer enthusiasm (and I do no want to hint possible commercial interests because the idea alone crosses a risky line), often it has played a secondary role in clinical trials, as stated by the authors.

Chapter 12, by Michelle J. Naughton and Sally A. Shumaker, catches the assessment of health-related quality of life, that is, trying to elucidate if the intervention improved, say, physical, psychological and social functioning, perception of well-being and health status, personal productivity, sexual life, sleep disturbances and other parameters. Not an easy task, indeed, although in certain extreme situations (as when life is supported by mechanical means and conscience is no longer present) may lead into deep controversies.

The remaining seven chapters deal, respectively, with participant adherence to the program, survival analysis, monitoring response variables (37 pages), issues in data analysis (38 pages full of statistical considerations), closeout (that is, how to properly terminate the study), reporting and interpreting of results (one of the most difficult steps because recommendations will be produced that may end up in massive use of the intervention), and finally a chapter devoted to multicenter trials.

The book provides an exceedingly good collection of references so that the interested reader can deepen in a particular subject. All in all, I would highly recommend it, to the physician, basic investigator and biomedical engineer. For the beginner, instead, it may be a little hard for some parts require good knowledge of statistics. It is no doubt excellent material for a course.

